# An On-Device Learning System for Estimating Liquid Consumption from Consumer-Grade Water Bottles and Its Evaluation

**DOI:** 10.3390/s22072514

**Published:** 2022-03-25

**Authors:** Avirup Roy, Hrishikesh Dutta, Henry Griffith, Subir Biswas

**Affiliations:** 1 Department of Electrical and Computer Engineering, Michigan State University, East Lansing, MI 48824, USA; royaviru@msu.edu (A.R.); duttahr1@msu.edu (H.D.); 2 Department of Electrical and Computer Engineering, The University of Texas at San Antonio, San Antonio, TX 78249, USA; henry.griffith@utsa.edu

**Keywords:** drink volume estimation, sip detection, embedded machine learning, on-device classification, neural networks, TinyML

## Abstract

A lightweight on-device liquid consumption estimation system involving an energy-aware machine learning algorithm is developed in this work. This system consists of two separate on-device neural network models that carry out liquid consumption estimation with the result of two tasks: the detection of sip from gestures with which the bottle is handled by its user and the detection of first sips after a bottle refill. This predictive volume estimation framework incorporates a self-correction mechanism that can minimize the error after each bottle fill-up cycle, which makes the system robust to errors from the sip classification module. In this paper, a detailed characterization of sip detection is performed to understand the accuracy-complexity tradeoffs by developing and implementing a variety of different ML models with varying complexities. The maximum energy consumed by the entire framework is around 119 mJ during a maximum computation time of 300 μs. The energy consumption and computation times of the proposed framework is suitable for implementation in low-power embedded hardware that can be incorporated in consumer grade water bottles.

## 1. Introduction

Ample hydration is a key to healthy living for people of all age groups. Inadequate water consumption can lead to a variety of health issues that include urinary tract infection, kidney failure, sticky mouth, headache, dizziness, electrolyte imbalance, tissue shrinkage, sunken eyes, and many more [1]. Severe lack of water intake can also affect the heat dissipation of the human body, resulting in feverish conditions. The effects of dehydration can be particularly adverse for the elderly population. These underscore the importance of appropriate hydration for a healthy lifestyle.

Many automated hydration-tracking and reminding technologies have emerged to improve individuals’ fluid consumption habits. These hydration-tracking systems can be categorized into wearable and non-wearable devices. The wearable devices mainly include wrist band-based systems [2] that monitor the fluid intake based on the wrist motion. The wrist band-based devices require an individual to always wear such a device and restricting the person to hold the water bottle/cup with the hand on which the device is worn. There are several fabric-based wearable devices to track different drinking and eating activities of a person [3]. Some prominent non-wearable approaches include the use of smart bottles [4]. These smart bottles can sense liquid level, bottle weight, and other parameters for estimating liquid consumption. However, there are reliability and durability issues since the level sensors require exposure in liquid for an extended period. There is also involvement of image-based approaches that measures liquid intake by analyzing bottle-tilt from phone-captured video, which is not user-friendly, as it also brings a threat to the user’s privacy by accessing a camera [5].

A common component across all these approaches is that on-bottle or on-cup (i.e., on-device) sensors are used for data collection during the drinking process. These data are then transferred through a wireless link to a connected phone or cloud-based entity for liquid consumption estimation. This approach enjoys the availability of ample off-device computing and memory resources, which are beneficial for complex detection and estimation tasks having a true positive rate for detecting a sip of around 98% [6]. However, it suffers from shortcomings such as heavy communication energy overhead and privacy concerns.

In this paper, we propose an on-device liquid consumption estimation framework. Two separate neural network models decide whether the data collected from an accelerometer are sips or not, and whether a sip in question is a first sip after a bottle is refilled, thus estimating the volume of fluid intake. The involvement of pre-trained and person non-specific neural networks ensures better data privacy since the no runtime user data are transferred to and from the device.

Based on this information, the consumed volume of liquid intake is estimated. An overarching goal in this approach is to avoid any raw data upload from a smart bottle to a phone or any other device. The final consumption estimation can be displayed on an on-bottle display. By doing away with any wireless link, the convenience and privacy of smart bottle users is improved.

The real-time estimation of the volume of consumed fluid is performed using a post-sip-detection unit. The volume estimation module detects the first sip intake after each bottle refill using a pre-trained neural network. The network uses information on maximum bottle inclination during a sip. From the detected first-sips and the known bottle volume, the system estimates cumulative volume of drink consumption using a self-correcting algorithm.

This work has the following scope and contributions. First, it develops low-complexity ML models for both sip detection and volume estimation tasks that are specifically optimized for embedded on-device implementations. This embedded setup overcomes the limited processing and memory resources together with energy constraints. Second, a detailed characterization of sip detection is performed to understand the accuracy–complexity tradeoffs by developing and implementing a variety of different ML models with varying complexities. Third, a predictive algorithm for consumed volume estimation is developed and implemented for its accuracy characterization. All these experiments are performed on a smart bottle created by integrating an embedded processor system on a regular grade water bottle.

## 2. Related Work

Embedded machine learning (ML) has been explored in many real-world applications, such as smart watches, sensors, hearing aids, etc. Most of the generic ML algorithms are computationally complex, thus leading to high power consumption and making them inapplicable for embedded applications with limited energy and memory budgets.

There are several papers such as [7] that aim at increasing embedded ML algorithm efficiency in terms of computational time and energy consumption by the means of an ML accelerator via decomposition of the most dominant high-dimensional operations, such as convolutions, matrix multiplications, and other methods of calculating the weights, into a series of vector reductions. A key challenge in the design of accelerators is to balance the tradeoff between efficiency and storage scalability, which by itself can be a challenge for implementation in embedded devices.

The work reported in [7,8] propose to increase computational efficiency of embedded ML algorithms by adopting various types of approximations at different levels including circuit, architecture, and algorithms. It has been observed that in most cases, the accuracy of the algorithms is heavily sacrificed to make it more efficient in terms of memory footprint and latency when these approximation methods are implemented.

The work described in [9] demonstrates on-hardware training of ML algorithm using customized data structure and computation sequence which relies on a Gaussian mixture model (GMM) that uses an expectation maximization (EM) algorithm with the minimum description length (MDL) criterion. This method of onboard training involves storage of large volumes of data, thus needing high memory usage. Moreover, onboard training and using the additional feature of the analytics services in [10] result in high power consumption. In this work, a low complexity machine learning framework suitable for low-power embedded hardware is utilized.

Fluid intake monitoring has primarily been done as questionnaire-based self-reporting with indirect estimation [10], which is usually not very accurate since it relies on manual and subjective inputs from the users. A few automated hydration tracking, and fluid-intake monitoring systems have recently been developed, which can be broadly classified into wearables and non-wearables.

Conventionally used wearable devices generally rely on a motion sensor fitted to the wrist of the subject with a help of a wrist band [2] that detects the motion of the bottle from the gestures of the wrist and can classify whether the bottle’s movement was that of a “fetch” or “sip”. The device has an inherent drawback that the person must always wear the band on the hand by which he/she holds the bottle; the sensor can also record gestures very similar to that of the sip movements, thus leading to loss of classification accuracy.

Among the non-wearable alternatives, there are systems that use vision-based approaches involving cameras and computer vision techniques coupled with deep-learning algorithms to track drinking activities [10] that can result in higher power consumption and a privacy threat.

There are a few commercial products available including the “Hidrate Spark” bottle by Apple Inc. [9] that uses a few on-bottle level sensors to monitor fluid intake subject, which are usually expensive and not long-lasting due to their constant exposure to liquid.

**Figure 1:**
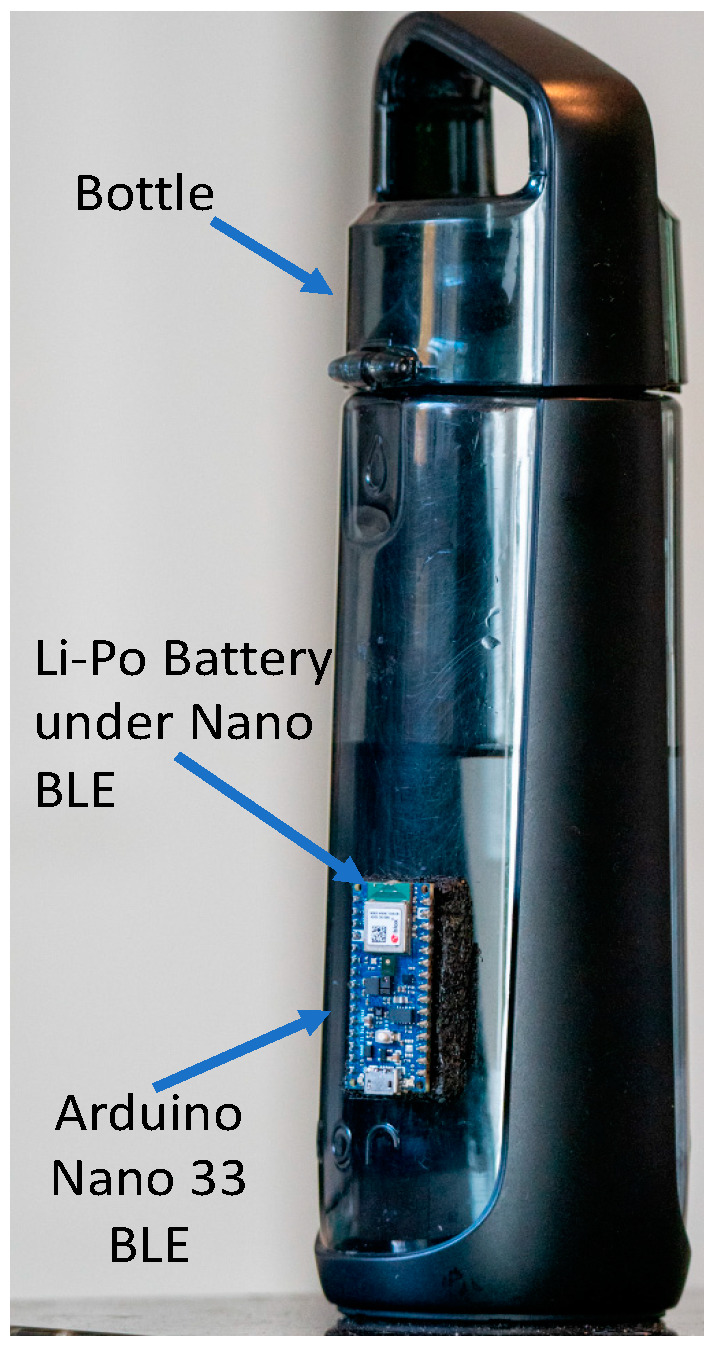
Instrumented bottle setup.

In [11], data are collected by a set of on-device accelerometers, and subsequently sent to an Android device using a Bluetooth link. Algorithms on the Android device classify the sips, and finally the consumption volume is estimated from the data collected. In [12], three IMU sensors were used to collect user gesture data from bottle movements, and a “temporal partitioning technique” was used to detect drinking windows from the time-series accelerometer data. Bluetooth-based data uploads in both the approaches are expensive, which is avoided in the system presented in this paper by running embedded ML algorithms on a bottle-attached microcontroller, as shown in Figure 1, thus making the overall system more energy efficient.

## 3. System Setup and Functional Overview

### 3.1. Bottle Instrumentation

The instrumented bottle setup is shown in Figure 1. A self-contained embedded microcontroller card, Arduino-based Nano 33 BLE Sense [13], is attached to a regular consumer-grade water bottle using Velcro. A small lithium-poly rechargeable battery is also provided (i.e., tucked underneath the Nano BLE card) to power the system. Nano BLE is a 3.3 V AI enabled board with dimensions of 45×18 mm. It comes with a set of embedded sensors including a nine-axis inertial sensor with a 120 Hz sampling rate, which is used extensively for this work. This board also consists of a microcontroller, nRF52840, from Nordic Semiconductors [14], and a 32-bit ARM Cortex-M4 CPU [15] running at 64 MHz. An ultralow power mode, suitable for embedded machine learning applications with power constraints, is a striking feature of this board.

For the system presented in this paper, all run-time neural network-based classifications are done within the Nano BLE card, which is pre-loaded with the NN models for both sip detection and volume estimation, as presented in Section 4 and Section 6. The models are trained on a PC using TensorFlow, which is then converted to a TensorFlow lite version and loaded on the Nano 33 BLE board for subsequent on-device classification.

Implementation of maximum energy economy would require a sleep–wake cycle in which the default state of the system would be sleep, and it would wake up only when any physical bottle movement is detected. Nano 33 BLE does not have an inbuilt idle/sleep mode. As a result, maximum energy economy is not implementable with this hardware. However, the research presented in this paper demonstrates how maximum energy economy can be achieved during the non-idle state when the actual sip and volume detection are executed. As a result, the research results from this paper are applicable for future commercial systems with hardware that supports the default idle/sleep state.

**Figure 2:**
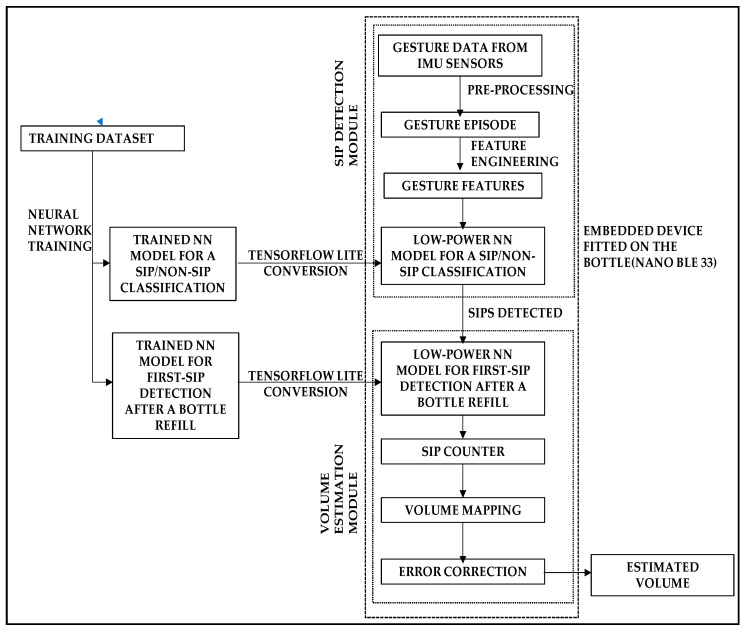
Block-diagram explaining the functionality and data/processing flow of the entire system.

Figure 2 depicts the entire workflow of the proposed system, which includes the machine-learning-based processing pipeline: data collection, pre-processing, feature engineering, neural-network-based sip classification, and neural-network-based consumption volume estimation. The processing pipeline is executed in the on-bottle embedded device from the gestures of the user’s motion while drinking.

### 3.2. Function Overview for On-Device Sip Detection

**Figure 3:**
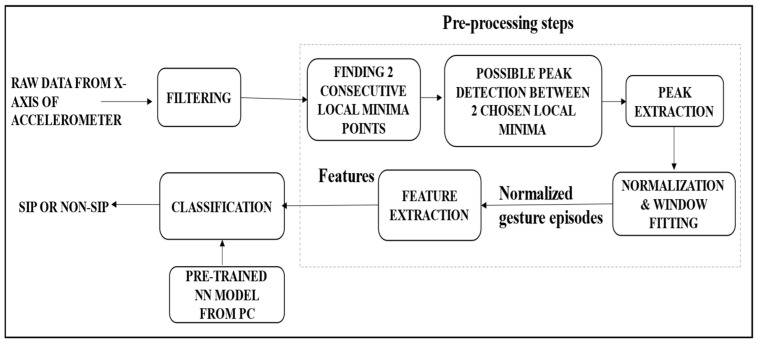
Schematic diagram of the proposed system for on-device sip detection.

The framework proposed in this paper possesses real-time ability to detect a sip, and subsequently to estimate the consumed volume of fluid from the number of sips and their specific features. All components of the end-to-end system for the sip detection module are shown in Figure 3. The embedded device (Nano 33 BLE) is fitted to the bottle in such a way (as shown in Figure 1) so that the *x*-axis of the accelerometer is aligned to the vertical axis of the bottle. To record any tilting gestures performed on the bottle, the *x*-axis data of the accelerometer [16], which is a part of the IMU sensors of the embedded device, is recorded. Since the tilt angle is used as the primary information for sip and volume detection, only the data from the *x*-axis of the accelerometer in the IMU sensors are used.

The accelerometer time-series data are first pre-processed in the on-bottle hardware where the peaks that can depict a possible sip are detected by finding one or more local maxima points between two consecutive local minima. Such peaks are extracted, normalized within a range of values, and fitted to a fixed length window. Features are then extracted from those fixed length windows, and subsequently fed into an on-device neural network (NN) classifier that classifies whether the activity performed on the bottle is a sip or non-sip. The on-device NN model is pre-trained on a PC and the model weights are stored on the embedded device’s memory to perform the on-device classification.

We used Google’s TensorFlow [17] to train neural network (NN) models on a PC. The trained models using the architectures as defined in Section 4.3.2 are first saved on the PC, and then optimized into a compressed version using TensorFlow Lite [18]. These compressed versions are optimized for low-power consumption embedded platforms. TensorFlow Lite models are meant for low-memory and low-processing platforms such as the Nano BLE 33 used in our system, which is implemented using the Arduino IDE platform [19].

## 4. On-Device Experimental Data Collection, Pre-Processing, and Classification

### 4.1. Data Collection

**Figure 4:**
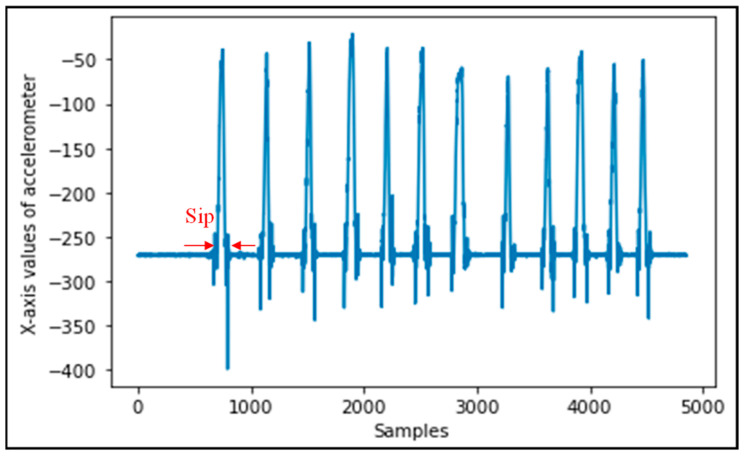
Representative data for 12 separate sip episodes.

To assure that the NN model loaded on the hardware makes accurate predictions, the data used for training the model incorporate different types of scenarios and activities. Instances of sip data are shown in Figure 4. Unlike for the sips, non-sip data can belong to many different subclasses resulting from different gesture scenarios performed on the bottle. Data were collected for non-sip scenarios including walking on flat ground with the bottle in hand, walking on stairs with the bottle held in hand, walking on flat ground with the bottle in a bag, walking on stairs with the bottle in a bag, keeping the bottle stationary in a constant position, and keeping the bottle in a constant position in a moving car and some in-hand fidgeting movements.

The objective here is to collect data from enough representative scenarios so that the NN training can be sufficiently general while classifying the non-sips.

For real-time testing, a combination of different activities, both sips and non-sips, was performed with the instrumented bottle, as shown in Figure 2. All experiments were performed by different subjects, each having specific signature drinking gestures. The number of different subjects used for data collection and the size of training and testing dataset are summarized in Table 1.

### 4.2. Real-Time Data Pre-Processing

*Filtering, Peak Detection, and Extraction:* The data pre-processing step begins with filtering the raw IMU sensor data using the moving average method [20]. For moving average, a consecutive window of 5 samples is used whose mean is calculated and used as the new sample. In this way, noise in the raw data is filtered out, which is useful in the subsequent stages of detecting peaks.

*Peak Detection and Extraction:* To detect a peak from the filtered data, two consecutive local minima points less than a threshold height Th are identified. Between those two local minima, if there exists one or more local maxima, then it is assumed that there exists at least one peak between those two local minima points. In that case, all the points between the local minima are extracted.

*Peak Normalization and Window Fitting:* All the points in the extracted peaks are normalized in the range 10 to 20 by using the equation below:

(1) $${\mathrm{norm}}_{x}=\left\{\left(\frac{x-\min\left(x\right)}{\max\left(x\right)-\min\left(x\right)}\right)+1\right\}\times 10$$

where ${\mathrm{norm}}_{x}$ = normalized values of the sample x, and min(x) and max(x) are the minimum and maximum values in the extracted peak episode, respectively.

The normalized samples of the extracted peaks are fitted in a window of a fixed size to have a constant length of episodes to be considered for classification of the sips and non-sips. Thus, each second of a gesture consists of 20 samples (accelerometer frequency is 20 Hz). A window of 120 samples is considered as an episode (maximum duration of a normalized sip: 6 s). Any episode with less than or more than 120 samples is padded with the value ”10” (minimum normalized value) at the end until the window length of episode becomes 120 samples or is pruned off, respectively. All classification operations are performed on such 120-sample data windows, which are referred to as the *episodes* for the rest of the text.

### 4.3. On-Device Sip Classification

To estimate the volume of fluid intake by a subject, the number of sips taken by the subject between two consecutive bottle refills needs to be detected with high accuracy. Thus, from the gesture data recorded by the embedded device fitted to the bottle, the gestures must be properly classified into the sips and non-sips classes. This classification is done by a trained neural network model for sip/non-sip classification. The NN model requires features extracted from the gesture signatures in order to classify the sip and non-sip classes accurately. Additionally, the neural network model needs to be lightweight in order to cater to the memory and energy limitations of the embedded device. Thus, an efficient feature engineering and selection of a lightweight NN model is highly required for the bottle-fitted embedded device.

#### 4.3.1. Feature Engineering

Five features representing each episode are extracted from the pre-processed data and are fed as inputs to an on-device neural network classifier. The features used for classification follow.

**Figure 5:**
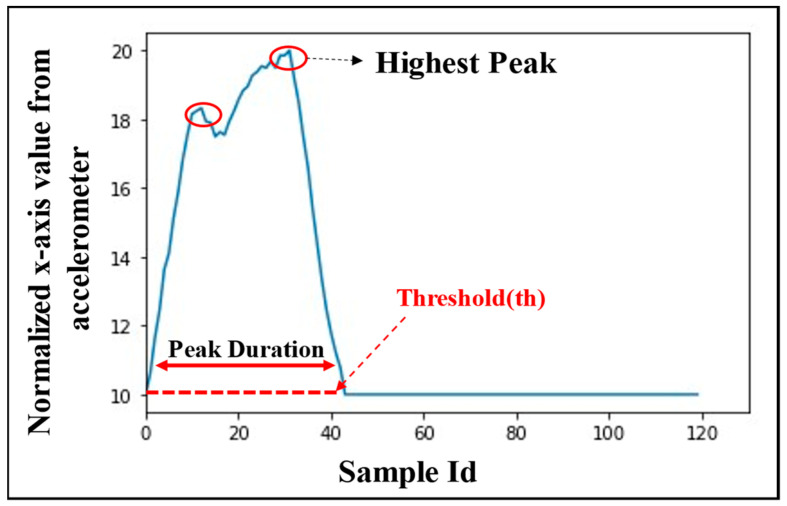
Sip/non-sip episodes explaining the classification features.

*Number of peaks in an episode:* It is calculated from the number of local maxima above a threshold value Th (normalized *x*-axis value from accelerometer as 10), which indicates the number of times a bottle is tilted during an episode. As an example, the number of peaks in the episode shown in Figure 5 is two (marked with red circles). The rationale behind using this feature is that the number of peaks in sip episodes are generally found to be less than those in non-sip episodes, although not always.

*Maximum peak height:* It is the height of the highest peak in a normalized episode, as shown in Figure 5. This feature represents the maximum tilt that the bottle experiences during an episode.

*Peak duration:* Peak duration represents the time in an episode for which the sample value remains above the threshold Th(10). This duration is generally found to be larger for sips as compared to non-sips.

*Number of samples within the stable portion of a peak*: Samples that are within 20% from the highest peak are counted and termed as the number of samples at the highest point in an episode. The higher this value, the larger is the probability of an episode being a sip.

*Height of the last point in an episode:* There exist some drinking episodes that are not completed within an episode of consideration. In such cases, the value of the last sample of the episode does not fall to the minimum normalized value in the episode. Usually for the non-sips, the value of this feature is the minimum normalized value in a gesture episode.

#### 4.3.2. Neural Network Architecture

In this proposed system, artificial neural networks (NN) [21,22] are used both to classify between sips and non-sips gestures, and to detect a first-sip after a bottle refill (Section 6.2). In order to cater to the limitations in memory and computational overheads of the bottle-fitted embedded device, selection of a proper NN architecture is very important. The NN selected must be such that the classification accuracy is high without having much complexity in compuatation and should not occupy much memory space on the device.

We experimented with a wide range of on-device NN architectures that were implemented within the on-bottle embedded device. Details of the architectures used for sip/non-sip classification are tabulated in Table 2. To keep the system computationally lightweight for the on-bottle embedded platform, the number of weights in the NN is attempted to be kept as low as possible. To accomplish this, performance is tested mostly for models with a single hidden layer, while changing the number of neurons in that layer. It is shown in Section 5 that a sip detection accuracy as high as 94% can be achieved with just one hidden layer. Experiments with a wider and deeper NN revealed that the performance of the NN does not improve by adding more neurons or hidden layers.

## 5. Experimental Results

### 5.1. Performance Accuracy

All the architectures from Table 2 are experimentally evaluated with the general NN features, as summarized in Table 3.

Sip/non-sip classification performance are captured in terms of true positive (tp) and false positive (fp) [23,24,25], where

(2) $$\mathrm{tp}=\frac{\mathrm{number}\;\mathrm{of}\;\mathrm{sips}\;\mathrm{corectly}\;\mathrm{detected}}{\mathrm{total}\;\mathrm{number}\;\mathrm{of}\;\mathrm{sips}\;\mathrm{actually}\;\mathrm{taken}}$$

(3) $$\mathrm{fp}=\frac{\mathrm{number}\;\mathrm{of}\;\mathrm{non}-\mathrm{sip}\;\mathrm{gestures}\;\mathrm{corectly}\;\mathrm{detected}\;}{\mathrm{Total}\;\mathrm{number}\;\mathrm{of}\;\mathrm{non}-\mathrm{sip}\;\mathrm{gestures}\;\mathrm{actually}\;\mathrm{taken}}$$

Additionally, an overall accuracy ŋ is measured as:

(4) $$ŋ=\frac{N_{\mathrm{sd}}+N_{\mathrm{nsd}}}{N_{s}+N_{\mathrm{ns}}}$$

where $N_{\mathrm{sd}}=$ number of sips correctly detected, $N_{\mathrm{nsd}}=$ number of non-sips correctly detected, $N_{s}=$ total number of true sips, and $N_{\mathrm{ns}}=$ total number of true non-sips.

**Figure 6:**
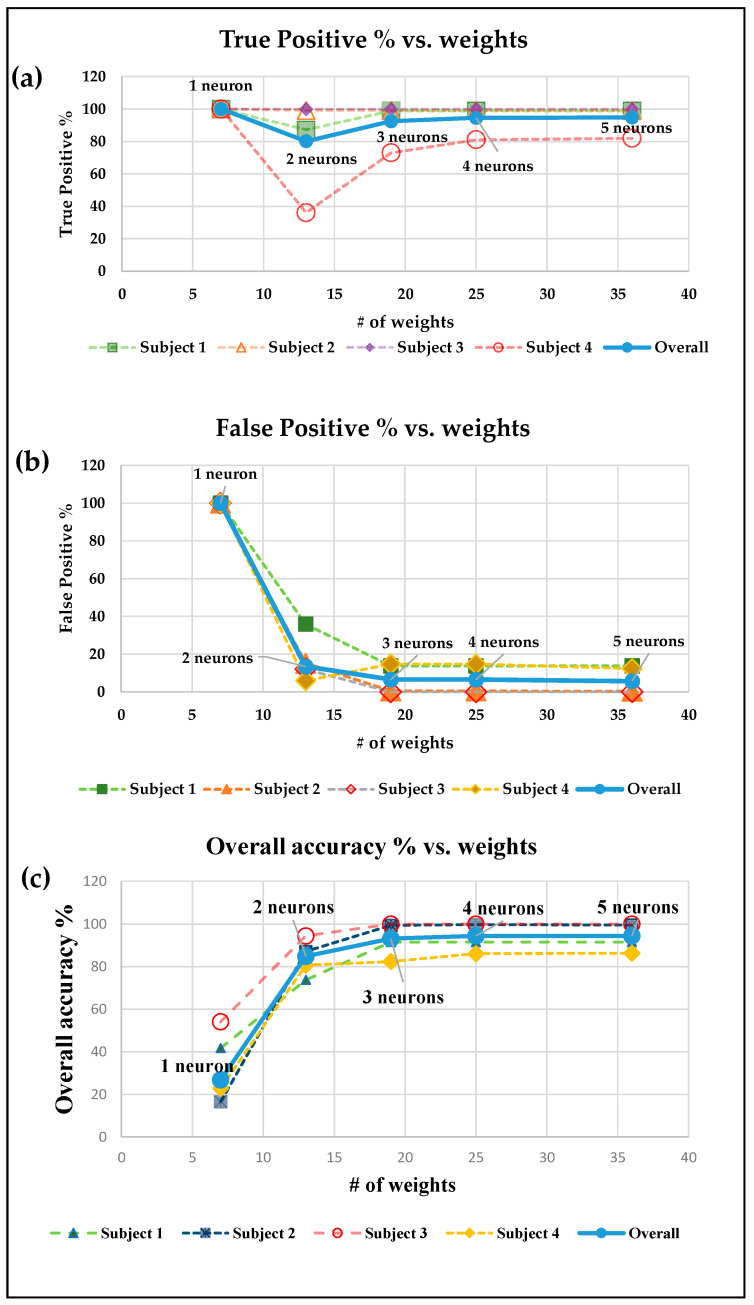
Sip detection performance results: (**a**) true positive vs. weights, (**b**) false positive vs. weights, and (**c**) overall accuracy vs. weights.

Each neural network architecture from Table 2 is tested with sip and non-sip episodes for four different subjects. The results for true positive %, false positive %, and accuracy for the one hidden layer model are reported in Figure 6a–c, respectively. These figures show the variation of the performance metrics with increase in the width of the hidden layer that leads to an increase in the number of NN weights. It can be observed from the figure that the classification accuracy increases with increase in the NN width, and it saturates at 94.8% for networks with four neurons in the hidden layer. False positive is maximum (100%) for the simplest model with one neuron, and it decreases with increase in the width of the only hidden layer. As the number of neurons in the hidden layer increases, the false positive decreases to 5% for the model with five neurons. On widening the only hidden layer with 20 neurons, the false positive does not change significantly. For true positive %, the neural network with one neuron gives 100% true positive, but false positive for that network is also 100%, which decreases the accuracy to 26%. Apart from this model, true positive also follows an increasing trend with increase in the width of the hidden layer. It should be noted that there is no increase in accuracy with the addition of hidden layers or neurons in the model.

### 5.2. Classification Energy Consumption

**Figure 7:**
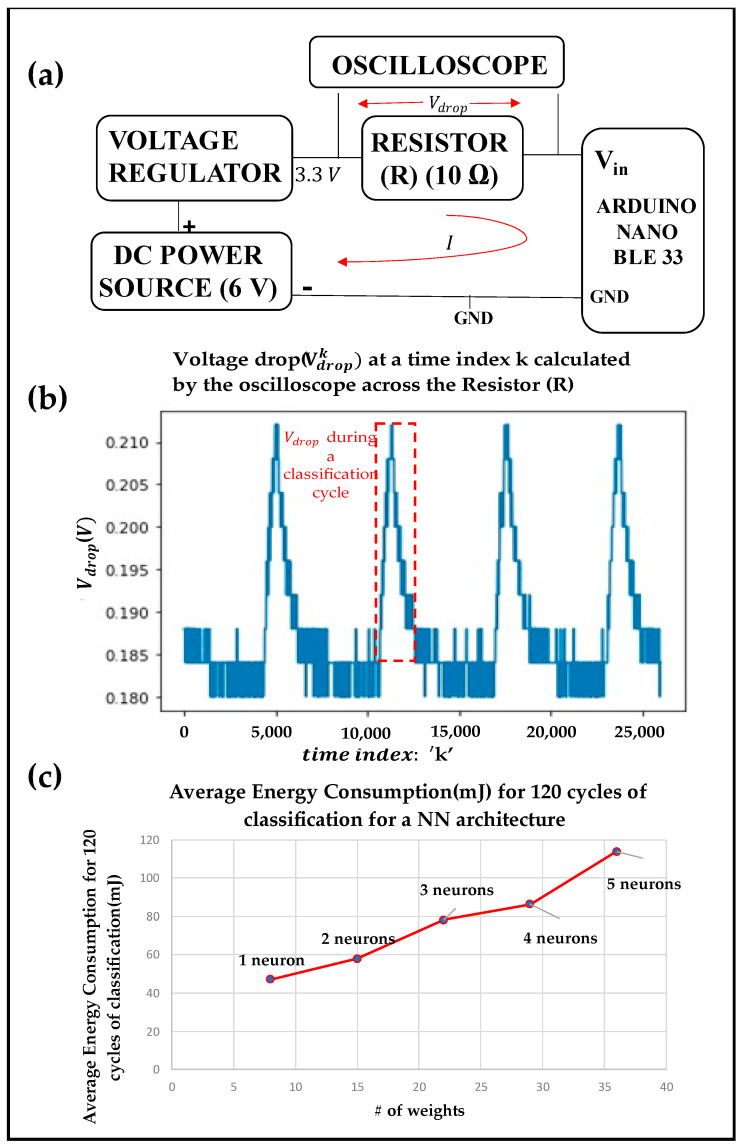
(**a**) Experimental setup to measure the energy consumption. (**b**) Voltage reading by the oscilloscope. (**c**) Average energy consumption (mJ) for each sip classification.

One of the important metrics for on-device embedded sip classification is the classification energy consumption. For measuring energy consumption, an arrangement as shown in Figure 7a has been arranged. Since Nano 33 BLE has a voltage limitation of 3.3 V, a voltage regulator is used to step down to 6 V power from the source to 3.3 V. A Tektronix 2024B Mixed Signal Oscilloscope is used for this experiment. The oscilloscope, with a sampling rate of 1470 Hz, is connected across the resistor to measure the voltage drop $V_{\mathrm{drop}}.$ The output signal from the oscilloscope is shown in Figure 7b, where each peak signifies the rise in voltage drop across the 10 ohms resistor (R) when the Nano 33 BLE performs the classification operation. Thus, each peak in the voltage-drop values shown in Figure 7b represents a classification operation by the Nano BLE device. The voltage drop values in a classification cycle are considered for the calculation of power consumption. The k-th sample of the input voltage ($V_{\mathrm{in}}^{k})$ in the Nano 33 BLE is calculated as:

(5) $$V_{\mathrm{in}}^{k}=3.3-V_{\mathrm{drop}}^{k}$$

where $V_{\mathrm{drop}}^{k}$ is the of voltage drop across the resistor R at $k^{\mathrm{th}}$ time index, read from the oscilloscope. Current ($I^{k}$) through the Arduino is computed as:

(6) $$I^{k}=\frac{V_{\mathrm{drop}}^{k}}{R}\;$$

Each sample has a sampling period (Δt) of 680 µs. The power ($P^{k})$ consumed by the device for performing classification operation at time index k is given by:

(7) $$P^{k}=V_{\mathrm{in}}^{k}.I^{k}$$

Thus,

(8) $$P^{k}=\left(3.3-V_{\mathrm{drop}}^{k}\right).\frac{V_{\mathrm{drop}}^{k}}{R}$$

**Figure 8:**
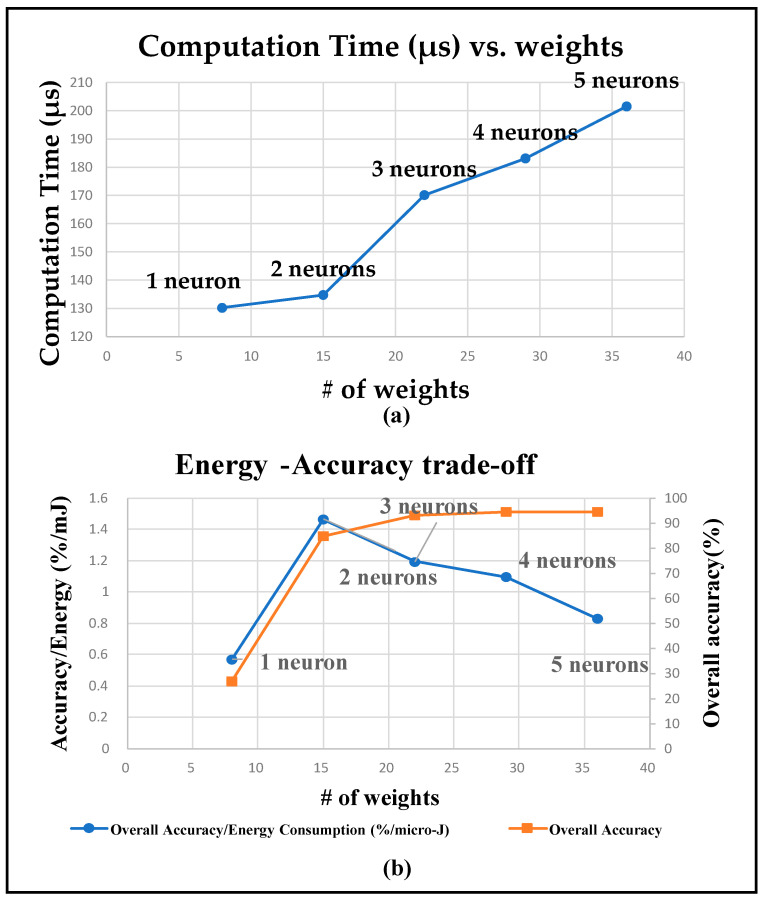
(**a**) Average computation time (µs) for each sip classification. (**b**) Energy–accuracy tradeoff.

The energy consumed during a classification cycle T is then computed as:

(9) $$E=\overset{T/\Delta t}{\underset{k=1}{\sum }}P^{k}\Delta t$$

where T is the classification computation time for a single cycle of classification, which is described in Figure 8a. To isolate the consumption only by the sip classification code, all other codes from the microcontroller are blocked during these experiments. For each of the NN models in Table 2, energy consumption is measured for 120 classification cycles, and the corresponding average is reported as a function of NN weight-counts in Figure 7c. The average energy consumption values reported in Figure 7c are computed from the $V_{\mathrm{drop}}^{k}$ values from the oscilloscope and using Equations (8) and (9). It can be observed that classifications take more energy in networks with wider hidden layers due to more computational overheads.

### 5.3. Classification Computation Time

Figure 8a reports sip classification computation duration for different network architectures. With increase in the number of neurons in the hidden layer, the number of network weights increases. This results in increase in number of computations and time taken by the hardware to carry out each classification operation.

### 5.4. Energy–Accuracy Tradeoff

Energy–accuracy tradeoff for different neural network architecture is presented in Figure 8b. The figure plots the ratio $\frac{\mathrm{Accuracy}}{\mathrm{Energy}\;\mathrm{Consumption}}\;\left(\%/\mathrm{mJ}\right)$ and the raw accuracy figures, both as a function of the number of weights. The ratio expresses how much sip detection accuracy is achieved for each unit of expended energy, which is a scarce resource in an embedded platform such as the one used in this work. It can be observed that $\frac{\mathrm{Accuracy}}{\mathrm{Energy}\;\mathrm{Consumption}}$ ratio is maximum for the network with two neurons in its hidden layer, and it falls with increase in the number of weights.

However, from the accuracy plot, it is seen that the sip classification indicates that the network that provides maximum accuracy per-unit energy does not provide an acceptable level of accuracy. For the model with three neurons in the hidden layer, the accuracy improves to 94%, but the $\frac{\mathrm{Accuracy}}{\mathrm{Energy}\;\mathrm{Consumption}}$ ratio falls by about 0.5%. This network provides the best balance between the acceptable accuracy and accuracy per unit energy, thus making it our network of choice. As a result, this network is used for the volume estimation mechanism presented in the next section. Note that for applications in which a lower accuracy is acceptable, the network with two neurons in its hidden layer can be a more appropriate choice from the standpoint of maximizing accuracy per unit energy expenditure.

## 6. Predictive Drink Volume Estimation

**Figure 9:**
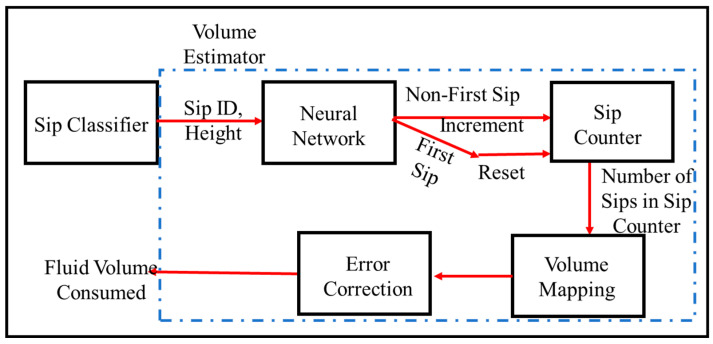
Algorithmic schematic of the drink volume estimation system.

Runtime drink volume estimation is performed using sip detections as described in Section 2, Section 3, Section 4 and Section 5. The algorithmic framework of the system is shown in Figure 9. The input to the volume estimator is a time series of the sip events along with the heights of the specific sip events. The height represents the maximum inclination of the bottle during a sip event. The first sip after a bottle refill requires the minimum amount of maximum inclination of the bottle during a sip. Leveraging this property, the system detects the first-sips since bottle refill. From the detected first-sips and the known bottle volume, the system estimates the cumulative drink volume, as shown in Figure 9. All the algorithmic details executed by each component of the schematic are given in Section 6.3.

This framework solves the problem of volume estimation directly using only bottle inclination information reported in [8,9]. The amount of bottle tilt cannot provide accurate volume intake estimation measure because the drinking pattern and the volume of fluid intake per sip vary from person-to-person. Here, the neural network models detect the sips and first-sips (i.e., after a bottle refill instance) using the extracted features from the accelerometer readings and the fluid intake volume is computed using that information. Additionally, the self-error correction mechanism after every first sip detected avoids the estimation error being accumulated. Thus, this framework provides a generalized and robust way of estimating fluid intake volume which is person non-specific.

### 6.1. Data Collection and Feature Extraction

For training, sip data were collected using the bottle mounted IMU sensor shown in Figure 1. In total, 300 sip events (12 per participant), labeled as a “first-sip” or “non-first-sip” after bottle refill, were recorded for five participants. For real time testing, experiments involving drinking events were conducted for the same five participants to determine accuracy–complexity tradeoffs.

(10) $$F_{1}\left(i\right)=p\left(i\right)-p\left(i-1\right)$$

(11) $${F\;}_{2}\left(i\right)=p\left(i\right)-p\left(i-2\right)$$

(12) $$F_{3}\left(i\right)=p\left(i\right)-p\left(i-3\right)$$

Here $F_{1}\left(i\right),{\;F}_{2}\left(i\right),{\;F}_{3}\left(i\right)$ represent three features as inputs to the neural network and p(i) is the sip height of the $i^{\mathrm{th}}$ sip from the sip classifier module.

All three features capture the temporal gradient of the sip heights (i.e., bottle inclination) in discrete time. The rationale behind using these features is that while drinking, the inclination of the bottle increases with decrease in fluid level. As a result, the sip height values increase with the increase in sip counts until the bottle is refilled.

These three features are computed each time a sip is detected, and the features are then used by the pre-trained neural network model stored in the device for detecting a first sip since bottle refill. The neural network gives decision on whether a sip from the previous module a first sip is or not. Based on this decision, the volume of fluid intake by the user can be estimated using the algorithm discussed in Section 6.3.

### 6.2. First-Sip Detection Using Neural Network

**Figure 10:**
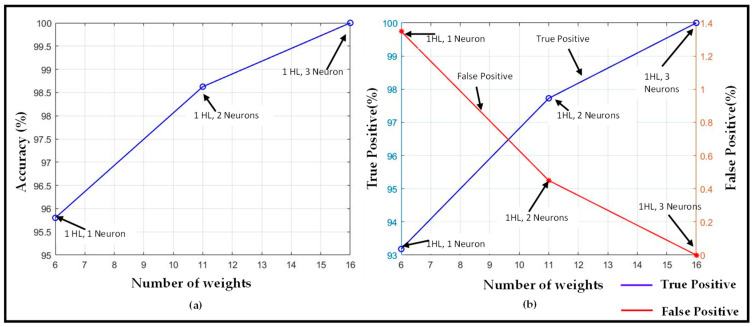
First-sip detection performance results: (**a**) Accuracy (%) vs. weights; (**b**) True Positive (%) and False Positive (%) vs. weights.

Similar to the sip detection part of the system, we retain the design goal of low classification–computational complexity, which is suitable for embedded on-device hardware. Experiments are performed with a one-hidden-layer model for varying number of neurons, and with general NN features summarized in Table 4. The first-sip detection results using that NN are presented in Figure 10. As expected, with an increase in the number of neurons (i.e., corresponding number of weights in the network), the overall accuracy and true positive rate increases. They eventually reach 100% for three neurons in the hidden layer. At the same time, the false positive rate decreases to zero. These results are noteworthy given the fact that such a high accuracy is achievable with an NN with a single hidden layer. The fact that such a high accuracy is achieved using only one hidden layer can be justified by the observation that the neural network for first-sip detection takes the input of time-series data of sip heights from the sip classification module. As explained in the prior sections, the sip detection accuracy of the sip classifier module is high (~95%). As the accuracy of the first-sip detection module depends on the sip classifying module, it is possible to achieve such high accuracy with only one hidden layer.

### 6.3. Volume Estimation

**Figure 11:**
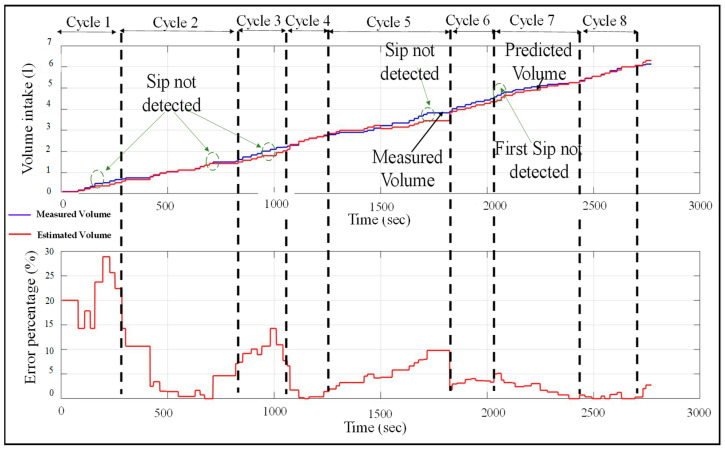
Predictive volume estimation and percentage error for 8 drinking cycles using 1 hidden layer and 2 neurons for the NN sip classifier.

Algorithm 1 describes the logic of cumulative volume estimation based on sip detection and the other conditions described above. The algorithm is executed upon each instance of a new sip detection, after which the current cumulative consumed volume is reported. These estimation results and their accuracies are reported in Figure 11.

As explained in Section 6.1, the volume estimation module takes time-series data of sip heights as inputs from the sip classifier module. The neural network takes the features computed using Equations (10)–(12) as inputs for detecting a first sip since bottle refill. For each sip detected as first-sip, all the sips prior to that are considered as the sips required to make the bottle empty. The number of such” non-first sips” is then mapped to the bottle volume V. The running estimation of volume per sip can be computed as $V_{\mathrm{Sip}}=\frac{V}{S_{\mathrm{count}}}$, where $S_{\mathrm{count}}$ is the number of sips prior to a first sip. This quantity is continuously updated as new sip events arrive from the sip detection module. For each incoming $i^{\mathrm{th}}$ sip from the sip classifier, the intake volume is updated as $V_{\mathrm{intake}}\left(i\right)=V_{\mathrm{intake}}\left(i-1\right)+V_{\mathrm{Sip}}$.

There can be certain scenarios when some sips are not detected by the sip classifier module, but to reduce the effect of these misclassifications, the algorithm uses a self-correction mechanism for every first sip detected by resetting the estimated volume for that drinking cycle equal to the known bottle volume.

This is done by computing the intake volume estimation error after each first-sip detected:

(13) $$V_{\mathrm{err}}=\min\left\{V_{\mathrm{intake}}\left(i\right)-V_{\mathrm{intake}}\left(i-S_{\mathrm{count}}\right)-k\times {\;V}_{\mathrm{bottle}}^{\mathrm{measured}}\right\},\;k\;\in I$$

**Algorithm 1** Predictive Volume Estimation1: **Input:** Time-series data of Sip heights (p(i) from Sip Classifier module // p(i) is the peak height of $i^{\mathrm{th}}$ sip
2: **Output:** Estimated Volume of intake fluid $(V_{\mathrm{intake}}$ 3: Initialize Sip Counter $S_{\mathrm{count}}$ 4. **for**
∀ sips with peak p(i) 5:    Compute features of p(i) 6:    Predict if p(i) is a first sip using Neural Network 7:    **if**
p(i) !=First Sip **then**: 8:          Increment $S_{\mathrm{count}}$ 9:          $V_{\mathrm{intake}}\left(i\right)=V_{\mathrm{intake}}\left(i-1\right)+V_{\mathrm{Sip}}$ //Estimate volume intake 10:          **end if** 11:  **if**
p(i)==First Sip **then** 12:          $V_{\mathrm{intake}}\left(i\right)=V_{\mathrm{intake}}\left(i-1\right)+V_{\mathrm{Sip}}$ //Estimate volume intake 13:          $V_{\mathrm{err}}=\min\left\{V_{\mathrm{intake}}\left(i\right)-V_{\mathrm{intake}}\left(i-S_{\mathrm{count}}\right)-k\times {\;V}_{\mathrm{bottle}}^{\mathrm{measured}}\right\},\;k\;\in I$ //Estimate error 14:          Update $V_{\mathrm{intake}}\;\left(i\right)=V_{\mathrm{intake}}\;\left(i\right)+V_{\mathrm{err}}$ 15:          Update $V_{\mathrm{sip}}=\frac{V_{\mathrm{intake}}}{S_{\mathrm{count}}}$ 16:    Reset $S_{\mathrm{count}}$ 17:    **end if** 18: **end for**

Here, $V_{\mathrm{bottle}}^{\mathrm{measured}}$ is the known measured volume of the bottle and $S_{\mathrm{count}}$ is the number of sips prior to a first sip.

The performance of the volume estimation module using Algorithm 1 is presented in Figure 11. The neural network for the sip classifier module used here has two neurons in its only hidden layer. The figure plots the estimated and true volume intake over a span of eight drinking cycles. The true volume indicates the volume intake calculated from the fill height of the bottle recorded after each drink. Here, each drinking cycle represents a period between two bottle refills. In the figure, the dotted lines separate two drinking cycles. The plot is updated with an increase in volume intake value each time a sip is taken by the user and/or a sip is detected by the sip classifier module. This indicates that the plot is updated only at certain time instances, which describe its stepped behavior. It can be observed that there are some sips that are not detected by the sip classifier module.

The sips not detected by the classifier are shown by the green circles in the figure. In those instances, where the sips are not detected, the estimated volume does not increase. This leads to an increase in the error percentage in volume estimation. However, because of the self-correction mechanism used by the volume estimator as described above, the error does not accumulate and reduces each time when a first sip is detected. Although the estimation error lies in the range 0–28%, the error reduces whenever a first sip is detected. This makes the system robust to the sip classification errors from the sip classifier. Note that for each first-sip detected, the estimation error does not always decrease to zero. This is because of the estimation error associated with the first-sip intake, that is, the difference between the estimated and true first-sip volume.

Note that the self-error correction mechanism of this framework of volume estimation works even in scenarios of missed first-sip detection or if the bottle is not fully refilled. This can be observed from Figure 11, where there is an instance of missed first-sip detection. In this case, the error accumulates until the estimated volume equals full bottle volume. After that, the estimated volume intake remains constant at the known bottle volume ($V_{\mathrm{bottle}}^{\mathrm{measured}}$) and the volume estimation error also stops accumulating until the next first-sip detection. Thus, this mechanism is robust to the scenarios where the bottle is partially filled, or the neural network model misses a first sip.

## 7. Summary and Conclusions

A lightweight liquid consumption estimation system is developed using an on-device neural network classification of user gestures while drinking from consumer grade bottles. The system first detects a sip from the bottle gestures recorded by the embedded IMU sensors using a pre-trained neural network model with a maximum accuracy of around 95%. The volume estimation framework detects the first sip after every bottle refill using another pre-trained neural network and estimates the volume intake by the user from the sip height of a detected sip. The predictive volume estimation framework also uses a self-correction algorithm that minimizes the estimation error after every fill-up cycle, thus reducing the effect of error from the sip-classification module. The system consumes a total energy of around 119 mJ to detect a sip and the volume consumed in that sip. Future work on this topic includes the development of volume estimation system in scenarios of limited labeled data availability using unsupervised and semi-supervised learning. Moreover, use of more energy-efficient hardware, with hardware sleep- and activity-based wakeup, can be included to reduce the power consumption by this fluid intake monitoring system; thus, detailed characterization of energy consumption using the mechanisms mentioned in [26] can be explored.

## Figures and Tables

**Table 1:** Training and testing datasets for SIP classification.

Training Dataset	
Number of sip episodes	1918
Number of non-sip episodes	1918
**Testing Dataset**	
Number of sip episodes	424
Number of non-sip episodes	1157

**Table 2:** Neural network models used for sip/non-sip classification.

Architecture Type	No. of Hidden Layers	No. of Inputs	No. of Neurons in the 1st Hidden Layer	No. of Neurons in the 2nd Hidden Layer	No. of Weights
1HL, 1N	1	5	1	0	8
1HL, 2N	1	5	2	0	15
1HL, 3N	1	5	3	0	22
1HL, 4N	1	5	4	0	29
1HL, 5N	1	5	5	0	36
1HL, 20N	1	5	20	0	141
2HL, 20, 15N	2	5	20	15	451

**Table 3:** Neural network architecture details.

**Model**	Sequential
**Layer Connectivity**	Fully Connected
**Optimizer**	SGD
**Loss Function**	Binary Cross-Entropy
**Epochs**	300
**Train–Validation Split**	80:20
**Batch Size**	100

**Table 4:** Neural network architecture details.

**Model**	Sequential
**Layer Connectivity**	Fully Connected
Optimizer	Adam
**Loss Function**	Binary Cross Entropy
**Training Epochs**	150
**Train–Validation Split**	80:20
**Batch Size**	10

## Data Availability

The accelerometer (*x*-axis) data used for training and testing are uploaded in the link: https://github.com/neewslab/Fluid-Intake-estimation-using-TinyML (accessed on 20 February 2022).
